# General practitioner-centered rural obesity management: Design, protocol and baseline data of the German HAPpEN pragmatic trial

**DOI:** 10.1016/j.pmedr.2024.102959

**Published:** 2024-12-26

**Authors:** Marika Haderer, Reiner Hofmann, Tina Bartelmeß, Laura König, Constanze Betz, Mirna Al Masri, Alisa Bader, Natascha von Schau

**Affiliations:** a Institute for Medical Management and Health Sciences, Project office of the Medical Campus Upper Franconia, University of Bayreuth, Bayreuth, Germany; b Faculty of Life Sciences: Food, Nutrition, and Health, University of Bayreuth, Kulmbach, Germany; c Faculty of Psychology, University of Vienna, Vienna, Austria; d Institute of General Practice, Friedrich-Alexander University Erlangen, Nuernberg, Germany

**Keywords:** Obesity, Primary health care, eHealth, Pragmatic trial, Social support, Rural

## Abstract

**Objective:**

HAPpEN aims to implement and evaluate a holistic general practitioner-centered, interdisciplinary obesity management strategy in rural Germany, focusing on feasibility, health outcomes, and economic benefits.

**Methods:**

HAPpEN is a 12-month, pragmatic single-arm, multicenter trial, informed by a formative survey, and initiated in April 2023 with 98 obese participants (body mass index, BMI ≥ 30 kg/m^2^) in Kulmbach, Germany. The program integrates nutritional counseling, physical activity, and behavior change techniques, including smartphone-based self-monitoring. Monthly consultations help set personalized goals using a multi-stage grading scale. Primary outcomes include BMI, body weight, waist circumference, heart rate, blood pressure and parameters, while secondary outcomes assess quality of life, wellbeing, health literacy, social interaction, and digital therapy support.

**Results:**

The baseline cohort (mean age: 46.9 ± 11.8 years, 74.1 % female) exhibited high obesity rates (mean BMI: 40.1 ± 6.1 kg/m^2^), with 48.5 % classified as grade III obese. Common comorbidities were hypertension (51.8 %), dyslipidemia (30.5 %) and diabetes (21.8 %). Chronic joint paint, mainly in the knees and hips, affected up to 82.4 %. A familial aggregation of obesity, diabetes, and cardiovascular diseases was noted, alongside behavioral challenges such as lack of physical activity (81.8 %) and unhealthy eating habits (56.8 %).

**Conclusion:**

HAPpEN addresses obesity's multifactorial nature through general practitioner-led, community-based, and digital strategies to promote sustainable lifestyle changes in rural areas. The trial aims to inform primary care obesity management guidelines, focusing on improving health literacy, patient engagement, and long-term clinical benefits. German Clinical Trials Register: DRKS00033916.

## Highlights

• pragmatic trial for rural obesity care, highlighting general practitioners' roles.
• general practitioners as central coordinators using psychological interventions.
• Evidence-based exercise and diet plans via a new smartphone-based grading scale.
• socio-cultural and rural challenges are tackled with community-based interventions.
• recruitment of at-risk population with prior unsuccessful weight loss attempts.

## 1 Introduction

Obesity is primarily associated with genetic disposition, poor diet and lack of physical activity (Mensink et al., 2013). In Germany, two-thirds of men and more than half of women are overweight (body mass index, BMI 25–29.9 kg/m^2^). Approximately a quarter of adults (23.3 % for men and 23.9 % for women) are classified as obese (BMI ≥ 30 kg/m^2^). Over the past two decades, the prevalence of obesity has significantly increased globally (Blüher, 2019). This trend not only leads to high healthcare costs but also poses significant health risks, including higher all-cause mortality (Durrer Schutz et al., 2019; NCD Risk Factor Collaboration and NCD-RisC)., 2021). Economic disparities between urban and rural areas modulate obesity prevalence (Blüher, 2019). Rural populations in the United Kingdom and Australia report lower engagement in leisure-time physical activity and fail to meet recommendations. Additionally, sedentary activities like viewing television and consumption of sugar-sweetened beverages occur more often contributing to higher obesity rates (Trivedi et al., 2015; Beverly, 2024; Borders et al., 2006). In Europe, obesity levels are elevated in the south compared to the north (Berghöfer et al., 2008), with similar trends observed in Germany, particularly in rural southwestern regions (Boehm et al., 2005). More detailed works regarding obesity and German rural environments are missing, highlighting the need for tailored guidelines and support for rural settings given limited access to health-related resources.

Effective multidisciplinary programs are essential for sustained obesity prevention and treatment involving nutrition, exercise, and behavioral changes into core medical healthcare (Durrer Schutz et al., 2019; Hauner et al., 2014; Lim et al., 2021). Due to their longstanding relationships with patients, especially in rural areas, general practitioners play a crucial role in incorporating socio-cultural aspects that allow tailored and comprehensive care (Whitlock et al., 2002; Van Dijk et al., 2006). Digitalization enhances obesity treatment additionally by enabling personalized programs and support that is time- and location-independent. In Germany, there are currently few long-term, general practitioner-centered obesity prevention and treatment programs, such as M.O.B.I.L.I.S., DocWeight, EndlichVital, Gesundheit PLUS, Bodymed and ACHT-Nachsorge (Hauner et al., 2014; König et al., 2018; Göhner et al., 2012; Berg Jr et al., 2008; Rudolph et al., 2016). These programs target individuals with a BMI ≥ 30 kg/m^2^ respectively ≥25 kg/m^2^ with increased comorbidity risk, offering nutritional, sport medicine, and behavioral components, but lacking digital therapy support. ACHT-Nachsorge includes digital tracking post-bariatric surgery. However, these programs do not fully integrate a multidisciplinary approach tailored to sociocultural contexts. HAPpEN aims to fill this gap with an interdisciplinary, evidence-based (Durrer Schutz et al., 2019; Hauner et al., 2014) holistic program focused on general practitioners and social support in a rural real-world setting. HAPpEN involves general practitioners as active therapists employing psychological interventions and providing personalized advice and education on nutrition and physical activity supplemented by a digital self-monitoring diary, fostering sustained behavior change. The primary objective is to enhance medical care for obese patients in rural settings, while the secondary objective is to evaluate the feasibility, health and economic benefits in daily life over 12 months. Here, we present formative survey data informing the HAPpEN pragmatic trial protocol, and corresponding baseline data, offering a detailed overview of our patient's sociodemographic and health-related characteristics. These data were collected through physical examinations, surveys, and questionnaires conducted in a rural setting. These data highlight the research value of such regions, capturing unique insights into patient's perceived causes of overweight and motivation for trial participation. The results aim to establish, evaluate, and optimize primary care-centered obesity management guidelines, particularly for non-urban regions.

## 2 Material and methods

### 2.1 Formative survey

Prior to designing HAPpEN, a survey was conducted to understand the eating and physical activity habits, food environment, health information sources, and use of digital health tools among German rural residents (König et al., 2024). The survey, administered in four family practices, collected data from 273 adults over a 10-week period from December 2022 to February 2023. The survey revealed a predominantly female population with a high prevalence of overweight and obesity among rural adults aged 40 and older. Participants reported preferences for home-cooked meals, limited local dining options, low exercise habits and high reliance on digital media, highlighting the need for (digital) accessible education and engaging, culturally relevant interventions (see Supplements, Supplementary Table 1–3). Consequently, to address these barriers and preferences, HAPpEN incorporates edutainment next to the general practitioner-centered approach to spruce up learning about nutrition and physical activity. This includes general practitioner-led teaching on nutrition and physical activity guidelines and community challenges facilitated through digital media like the HAPpEN app and website with integrated exchange forum. Additional healthy recipes, in-person-edutainment activities and physiotherapist-led exercise groups were included (see Supplementary Table 3 and section 2.4).

### 2.2 Design

HAPpEN is a pragmatic single-arm trial in six rural primary care offices in Kulmbach, Germany, supported by nine physiotherapists for exercise interventions. Data are collected only from patients who provided written consent, with the option to withdraw anytime. The data collection process included physical examinations, surveys, and self-reporting questionnaires. Comparative baseline data are drawn from population-based statistics in Kulmbach. All procedures were performed in compliance with relevant laws and guidelines and have been approved by the ethical institutional committee of the University of Bayreuth (Az. O 1305/1 – GB, April 2023). The trial is registered at the German Clinical Trials Register (DRKS00033916).

### 2.3 Participants

For recruitment, general practitioners informed patients during routine consultations, posters were distributed in clinics and a radio advertisement was aired shortly before the trial. Patients aged 18–65 years, meeting the following inclusion criteria were enrolled in the trial:

• Confirmed diagnosis of obesity (BMI ≥ 30 kg/m^2^) at the time of recruitment
• Sufficient knowledge of the German language
• Ability and willingness to use a digital, app-based self-monitoring diary

Patients fulfilling any of the following criteria were excluded from the trial:

• Condition of incapacity for load bearing, e.g. following a surgical procedure
• Patients requiring urgent major surgical intervention
• Patients with seizure disorders or epilepsy
• Dementia/cognitive deficit, which contradicted with the program
• Psychiatric illness contradicting the program
• Participation in another study (up to one month before start timepoint)
• Heart failure from New York Heart Association class III onwards
• Uncontrolled arterial hypertension
• Severe pulmonary comorbidity (severe asthma, chronic obstructive pulmonary disease, modified Medical Research Council dyspnea scale >2 / chronic obstructive pulmonary disease assessment test >10, pulmonary fibrosis)
• Severe psychiatric eating disorders
• Other severe illness that rendered physical exertion impossible

The intervention targeted an area-based sample within the district of Kulmbach, focusing on the towns of Marktleugast, Presseck, Untersteinach, Thurnau, Kasendorf and Stadtsteinach. The rural population (except Untersteinach) was defined as participants from sparsely populated rural areas, characterized by low population density, a predominantly agricultural landscape and limited infrastructure (BBSR, 2024; Küpper, 2016).

### 2.4 Guideline-compliant obesity management intervention

#### 2.4.1 Overview of intervention components

HAPpEN is a multi-component intervention, drawing on face-to-face and digitally delivered content and promoting social support and knowledge exchange. It involved general practitioners as the primary point of contact and guide through the intervention, as well as nutrition and behavior change experts and physiotherapists. The intervention components and related behavior change techniques (Michie et al., 2015) are listed in Table 1 and are described in more detail in the following.

**Table 1:** List of HAPpEN intervention components and implemented behavior change techniques.

HAPpEN intervention component	Brief description	Behavior Change Techniques (BCT taxonomy v1) (Michie et al., 2015)
Individual goal setting and planning	Step-wise, tailored goal setting across 17 weight management and health topics (e.g. diet, physical activity, sleep), with participants selecting two to three topics/month and choosing from five to eight goal levels, this is performed via the HAPpEN app and during monthly sessions led by general practitioners	1.1 goal-setting (behavior), 1.5 review behavior goal(s), 1.6 discrepancy between current behavior and goal, 2.2 feedback on behavior, 8.7 graded tasks
Self-Management	Using action and coping planning, participants identify practical strategies for daily implementation and potential barriers, developing individualized plans to enhance adherence and manage challenges effectively	1.2 problem-solving, 1.4 action planning
Problem Solving/ Feedback	Withing general practitioner-led monthly sessions, focus on eliciting feedback, addressing personal barriers, and fostering self-reflection to enhance intrinsic motivation and support behavior change, using motivational interviewing techniques	1.2 problem-solving, 2.1 feedback on behavior, 2.7 feedback on outcome(s) of behavior
Self-monitoring	Enabling self-monitoring of dietary and activity goals, weight progress curves and personalized training plans via HAPpEN app. Weight and BMI^1^ are also tracked at general practitioner practice every month.	2.3 self-monitoring of behavior, 2.4 self-monitoring of outcome(s) of behavior
Gamification	Challenges and learning units, provided digitally via the app and forum. These interactive tools incorporated regional settings and daily routines to engage participants in adopting a health-promoting lifestyle through playful and practical approaches	4.1 instruction on how to perform a behavior, 10.3 non-specific reward
Education	Providing evidence-based guidance on diet, physical activity and behavior change strategies in teaching session. General practitioner-led monthly sessions with integration of motivational interviewing and nutritional counseling	4.1 instruction on how to perform a behavior, 4.2 information about antecedents, 5.1 information about health consequences, 6.1 demonstration of the behavior, 8.1 behavioral practice/ rehearsal, 9.1 credible source
Social interaction	Facilitated through physiotherapists-led exercise group session, a peer exchange forum, and in-person activities (cooking and hiking events and barbecues), promoting social support and engagement in a health-oriented community	3.1 social support (unspecified), 3.2 social support (practical), 4.1 instruction on how to perform a behavior, 12.2 restructuring the social environment

^1^BMI, body mass index

#### 2.4.2 Assessments by and consultations with the general practitioners

**Fig. 1:**
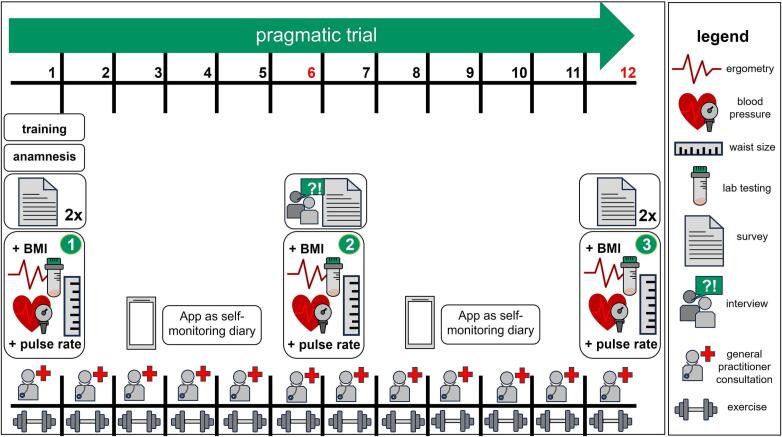
Timeline of the 12-month pragmatic, single-arm HAPpEN trial from the patient's perspective. At the start, next to nutrition and behavioral education, a comprehensive medical history is taken including detailed anamnesis via surveys and a standardized physical examination (body height, weight respectively body mass index (BMI), waist circumference, blood pressure, heart and pulse rate, joint function, thoracic auscultation, abdominal examination, motor and sensory tests), supplemented by ergometry and lab tests to assess patients' fitness level and health status. Monthly appointments with general practitioners and group sessions with physiotherapists support patients in identifying performance goals and increasing muscle-strengthening exercises or physical activity in general. The HAPpEN app acts a stepwise digital diary, allowing for education and monthly nutrition and exercise goals to be set, aided by video-based training. Follow-up physical examinations are conducted at six and 12 months. Additionally, patients' perceptions of social group interactions, dynamics and social media usage are continuously assessed and documented through surveys and interviews.

HAPpEN is set up in accordance with the 5 A model and the S3 Guideline for Obesity Prevention and Therapy (Hauner et al., 2014) (see Supplementary Table 4). In the first ASSESS step, eligible patients willing to change weight are identified. At enrollment, a comprehensive medical history is obtained, including a standardized physical examination, ergometry, lab testing, and self-report surveys to evaluate fitness, health status and body weight management. In addition, to prevent complications and sensibilize for their own health condition, patients undergo examinations by doctors after six and 12 months (see Fig. 1 and Table 2). Second, in the ADVICE step, patient-specific problems, causes, and obstacles to weight reduction are recorded for individual education. A therapy plan involving nutrition, exercise, and behavioral techniques is developed, tailored to the patient's initial weight, comorbidities, and weight loss goals. Third, in the AGREE step, individual goals are set step by step to be achieved within every four weeks and evaluated and discussed in the monthly appointments. Fourth, during the ASSIST step, obstacles to achieving agreed goals are evaluated. Finally, in the ARRANGE step, general practitioners use motivational interviewing techniques (Barrett et al., 2018) to sustain participants' motivation, assessing current resources and circumstances to support lifestyle changes beyond the trial.

**Table 2:** Data Collection within the 12-month pragmatic HAPpEN trial.^2^

	Enrolment	Pragmatic trial (months)										Trial End	Instrument	Statistics
Timepoint	1	2	3	4	5	6	7	8	9	10	11	12		
Social anamnesis	x												questionaire	Person‘s-chi-squared test
Body weight management	x												questionaire	
Wellbeing/quality of life/sleep	x/x/x^3^					−/(x)/x						x/x/x	questionaire	Analysis of Variances, t-test
Medical history (smoking, joint trouble)	x					x						x	questionaire	Cochran's Q test, Friedmann test
BMI^1^, weight, waist circumference, blood pressure, blood test, thorax-, abdomen-, neurological tests, ergometry	x					x						x	medical examination	Analysis of Variances
Medication	x					x						x	questionaire, medication plan	Cochran's Q test
Family history and diseases	x												questionaire	
Nutrition literacy eating/drinking behavior	x					x						x	questionaire	Cochran's Q test
Physical literacy and activity	x					x						x	questionaire	Cochran's Q test
HAPpEN-App												x	questionaire	t-test, correlation, regression
Technical affinity	x												questionaire	
Social support/social network	x					x						x	questionaire/ interviews/	
General practitioner-centered program						x						x	questionaire/ interviews	

^1^BMI, body mass index

^2^Data will be collected at indicated time points using shown measurement instruments. Statistical evaluation is performed utilizing the specified test.

^3^The consecutive Xs represent the individual assessments of wellbeing, quality of life, and sleep at the indicated time points. If three Xs are present, all three assessments were conducted at that time point; if only two Xs are present, only two assessments were performed accordingly.

#### 2.4.3 Recommendations for diet and physical activity

Instead of focusing on calorie counting, participants receive nutrition guidance, mainly based on the mediterranean diet. Emphasis is placed on consuming foods with low energy density (Stelmach-Mardas et al., 2016). For physical activity, recommendations focus on daily walking, moderate-to-vigorous physical activity, and strength exercises (World Health Organization, 2020). Finally, sleep is discussed as an important additional health behavior.

Step-wise goals across 17 topics (see Supplementary Table 5) related to weight management and health promotion (Schlesinger et al., 2019; *Forschungs und Prax der Gesundheitsförderung. Published online*, 2017) are designed to be challenging, achievable and tailored to individual performance and circumstances (Quintiliani et al., 2014). Topics cover areas such as reducing soft drinks, snacks and fast food, increasing plant-based protein, physical activity, and daily steps, improving sleep behavior and reducing sedentary time. Participants are advised to tackle two to three topics each month. Within each topic, participants are asked to select a challenging goal out of a list of five to eight options ranging from “I am not aware of how to address this topic in my daily life” (with the aim to track their behavior to identify areas for change) to “reduce bad habits and improve lifestyle up to six to seven days/week”. General practitioners assist with background information and goal setting as needed.

#### 2.4.4 Educational sessions led by nutrition and behavior change experts

In three 90-min sessions at accessible located venues, nutrition and behavior change experts provide evidence-based guidance on diet, physical activity and behavior change strategies. These sessions focus on the rationale, practical application and techniques like step-wise adherence, action and coping planning, grounded in cognitive behavioral theory.

#### 2.4.5 Activities led and supported by physiotherapists

HAPpEN involves monthly physiotherapists-led group sessions tailored to participants' fitness levels and local accessibility. These sessions teach safe, effective exercise for home or group practice, minimizing injury risk and supporting consistency. Animated tutorials in the app supplemented home practice, allowing physiotherapists for personalized adjustments based on participants' feedback.

#### 2.4.6 Digital website and app for self-monitoring and social support

**Fig. 2:**
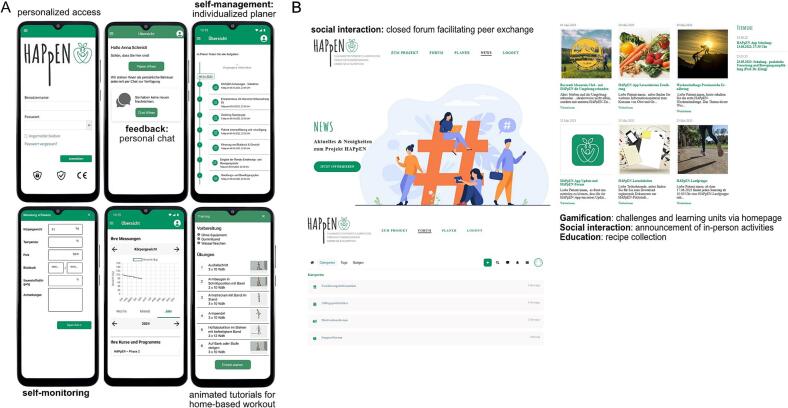
Screenshots of the HAPpEN app (A) and homepage (B) as integrated digital support tools in HAPpEN. (A) The HAPpEN-app functioned as a personalized patient diary, facilitating feedback, self-management and -monitoring. It included a planner for goal setting and weight tracking, with automatically generate overviews (e.g. weight trajectory curves) based on user input to provide feedback on weight management. (B) The HAPpEN homepage page offered educational content on health-promoting lifestyles, accessible via news page but also via app. Additionally, it featured weekly edutainment challenges, announcements for social events, and a forum for participant exchange.

The HAPpEN app and website provides tools for self-monitoring, including tracking dietary and activity goals, weight progress and personalized training plans with animated tutorials (see Fig. 2). Participants can access recipes, educational modules and challenges to support a healthy lifestyle with direct communication through the app-linked chat function for consultation with physicians and physiotherapists. A closed forum facilitates peer exchange on tips, challenges, successes, and event announcements. Initial training and ongoing support promote effective app usage.

#### 2.4.7 Additional in-person opportunities for social support

Social support was further provided via monthly sessions, as well as regionally accessible cooking and hiking events, promoting engagement and community-building.

### 2.5 Measures

Primary outcomes include BMI, body weight, waist circumference, blood parameters (e.g. Hemoglobin A1c, lipid metabolism and uric acid), blood pressure and heart rate. Secondary outcomes comprise:

• Quality of life (Morfeld and Bullinger, 2008), wellbeing, anxiety (Spitzer et al., 1999) and disease burden (e.g. medication, comorbidities, sleeping behavior)
• Nutrition, health and physical activity literacy as well as health behavior change for an improved understanding of patients' personal health status (Spitzer et al., 1999; Westenhöfer et al., 2003; Bull et al., 2009; Pfeiffer et al., 2020)
• Benefit and effectiveness of social interaction (Borek et al., 2019; Carrillo-Álvarez et al., 2019; Nackers et al., 2015)
• Acceptance, efficiency, user-friendliness and -experience of digital therapy support by evaluating self-reported application usage frequency and its most relevant features (Cheah et al., 2022; Laugwitz et al., 2008; Sekhon et al., 2022)
• Economic evaluation through literature-based modeling of the costs and their assessment relative to the generated benefits

### 2.6 Statistics

BMI and body weight will be compared between the patients in the trial and those receiving current obesity standard care. Furthermore, outcomes will be analyzed at baseline and after six and 12 months, using paired *t*-tests, analysis of variances, Cochran's Q or Friedman test (see Table 2). Data distribution will be analyzed using Shapiro-Wilk normality test and histograms.

## 3 Results

### 3.1 Baseline demographic characteristics of the trial population

The baseline population consisted of 98 German participants with a mean age of 46.9 ± 11.8 years, slightly higher than the national average and average from the federal stat of Bavaria (Statistische Bibliothek, 2022). Most participants were female (74.1 %) and married, cohabitating with their spouse (64.3 %) (see Table 3). Compared to national averages, higher proportions had secondary education (43.5 %) and professional training (62.2 %), but fewer held high school diplomas (20.0 %) or university entrance qualifications. The majority (83.3 %) were employed, with 14.5 % working in shift-based jobs.

**Table 3:** Demographic characteristics and weight history of the baseline population (98 German adults), assessed at the start of the HAPpEN trial in May/June 2023.

Demographic characteristics	baseline population	Weight history	baseline population
**Gender** Female/Male	74.1 % (63) / 25.9 % (22)	**Childhood Overweight**	59.8 %
**Age**	46.9 ± 11.8 years (83)	**Previous Weight Loss Attempts**	96.6 % (84)
**Practice Affiliation** Oberlandärzte Marktleugast Seitter/Tischer Presseck Seitter/Tischer Untersteinach Seitter/Tischer Thurnau Ipta Kasendorf Olszewski Stadtsteinach	52.0 % (51) 5.1 % (5) 6.1 % (6) 16.3 % (16) 13.3 % (13) 7.1 % (7)	**Years Spent on Weight Reduction** 10 years five to nine years two to four years < two years	67.1 % (55) 18.3 % (15) 7.3 % (6) 7.3 % (6)
**Marital Status** Single, living alone Single, living with a partner Married, living with spouse Married, living separately Divorced Widowed	13.1 % (11) 14.3 % (12) 64.3 % (54) 1.2 % (1) 6.0 % (5) 1.2 % (1)	**Causes/Triggers of Weight Gain** Life changes: career change, relocation, retirement Changes in family environment: pregnancy, children, marriage, divorce Decreased physical activity Unbalanced diet Menopause/hormonal changes Medications/underlying health conditions Increased alcohol consumption Smoking cessation Other: stress, fieldwork, acute illness/injury, bullying, exam situations	13.6 % (12) 51.1 % (45) 63.6 % (56) 58.0 % (51) 28.4 % (25) 34.1 % (30) 5.7 % (5) 17.0 % (15) 13.6 % (12)
**Education** No formal qualification Basic education Secondary education High school diploma	1.2 % (1) 35.3 % (30) 43.5 % (37) 20.0 % (17)	**Reason for planned weight loss** Improved well-being and body image Life changes Improved health status Pain reduction Improved physical fitness/muscle building Other: engaging in hobbies, role model for children	96.6 % (85) 28.4 % (25) 89.8 % (79) 51.1 % (45) 78.4 % (69) 3.4 % (3)
**Vocational Training** No qualification Apprenticeship/vocational training Technician/Master's/Further education University of Applied Sciences/ University degree	6.1 % (5) 62.2 % (51) 23.2 % (19) 8.5 % (7)	**Barriers to Weight Management** Strong physical hunger Eating behavior Psychological issues: depression, attention-deficit-hyperactivity disorder, attention-deficit disorder, psychosis, eating disorders Health issues: underlying conditions, pain-related limitations Lack of physical activity Insufficient time or stress in daily life Societal, private, and professional obligations Other: lack of motivation, medication use, shift work	19.3 % (17) 56.8 % (50) 21.6 % (19) 26.1 % (23) 81.8 % (72) 56.8 % (50) 56.8 % (50) 11.4 % (10)
**Employment Status** Retired Unemployed Employed Student	9.5 % (8) 6.0 % (5) 83.3 % (70) 1.2 % (1)	**Previously Used Weight Loss Methods** Dietary changes/diets: e.g., calorie counting (with app), Zanadio app, Weight Watchers, intermittent fasting, shakes, “Losing Weight with Pleasure,” Oviva app, Bonvita, “Slim in Your Sleep” Physical activity: rehabilitation sports, fitness centers Behavioral therapy: e.g., self-monitoring, acquiring nutritional competence, Weight Watchers, Zanadio app, Oviva app Social reinforcement: e.g., Weight Watchers – group therapy, Zanadio app – counseling, Oviva app – counseling, psychotherapy, patient groups, family support Medications	89.4 % (76) 30.6 % (26) 34.1 % (29) 37.6 % (32) 2.4 % (2)
**Working Hours**	32.5 ± 10.4 h/week (75)	**Weight Loss Achieved by previous methods**	91.5 % (75)
**Shift Work**	14.5 % (11)	**Occurrence of Yo-Yo Effect following previous methods**	91.8 % (78)
**Smoking Status** Current smoking status (years / cigarettes/day) Former smoker Duration of smoking cessation (years)	20.0 ± 12.1 (8) / 18.0 ± 10.6 (9) 35.6 % (31) 13.9 ± 9.9 (31)	**Weight Monitoring** Daily Three times/week One to two times/week once/week never	12.8 % (11) 15.1 % (13) 26.7 % (23) 33.7 % (29) 11.6 % (10)

### 3.2 Baseline health characteristics

A significant portion of participants (59.8 %) reported being overweight during childhood, and 63.6 % identified decreased physical activity as a key factor in their weight gain next to poor dietary habits (58.0 %) and life changes such as pregnancy, marriage, or job transitions (51.1 %). Additionally, hormonal changes, particularly during menopause, were cited by 28.4 % of participants. The main motivators for participants' weight loss efforts were to improve overall well-being and body image (96.6 %) and to enhance physical health (89.8 %). Many participants also sought to reduce pain (51.1 %) and increase physical fitness or muscle strength (78.4 %). Some aimed to be role models for their children or to better engage in hobbies (3.4 %).

Most (96.6 %) participants had previously attempted weight loss, with 67.1 % of participants indicating more than 10 years of experience in weight management efforts. The most common methods included dietary changes (89.4 %), physical activity (30.6 %), and behavioral therapy (34.1 %). However, 91.8 % experienced weight regain. The most frequently reported barriers to successful weight management included lack of activity (81.8 %), challenges with eating behaviors (56.8 %), and physical hunger (19.3 %). Psychological factors, such as depression and other mental health conditions, were reported by 21.6 %, and 26.1 % indicated health-related problems that limited their ability to exercise (see Table 3).

Medical history revealed a high prevalence of obesity-related comorbidities. The mean BMI was 40.1 ± 6.1 kg/m^2^ (see Table 4), with 48.5 % classified as having grade III obesity (BMI > 40) compared with the national obesity rate of 23.3–23.9 % (Blüher, 2019). The mean baseline waist circumference was 123.6 ± 15.2 cm, indicating a high level of visceral fat among participants and corresponding to the high prevalence of hypertension (51.8 %), diabetes mellitus (21.8 %) and dyslipidemia (30.5 %). Further conditions included sleep apnea (9.4 %), gastroesophageal reflux disease (27.1 %), and osteoarthritis, particularly in the knees (25.0 %). Additionally, 20.5 % of participants were diagnosed with fatty liver disease, and 10.7 % reported joint-related diseases such as rheumatism.

**Table 4:** Medical and family history and joint complaints of the baseline population (98 German adults), assessed at the start of the HAPpEN trial in May/June 2023.

Medical history		baseline population
**Weight**		117.5 ± 22.0 kg (98)
**Height**		170.6 ± 7.5 cm (97)
**Waist circumference**		123.6 ± 15.2 cm (88)
**BMI**^**1**^ Obesity Grade I (BMI 30.0–34.9) Obesity Grade II (BMI 35.0–39.9) Obesity Grade III (BMI >40)		40.1 ± 6.1 kg/m^2^ (97) 22.7 % (22) 28.9 % (28) 48.5 % (47)
**Blood Pressure (systolic/diastolic)**		134.8/85,6 ± 13.7/9,0 mmHg (93)
**Heart Rate**		81.0 ± 12.9 beats/min (94)
**Comorbidities** Hypertension Diabetes Mellitus Reflux Dyslipidemia Uric acid metabolism disorder Myocardial infarction Vascular diseases Lung diseases Sleep apnea Hypothyroidism Kidney diseases Urinary incontinence Gallstone disease Chronic inflammatory bowel disease Fatty liver Joint diseases/rheumatism Knee osteoarthritis Hip osteoarthritis Spinal disc herniation Cervical disc herniation Thoracic disc herniation Lumbar disc herniation Degenerative spine syndrome Degenerative cervical spine disease Degenerative lumbar spine disease Shoulder syndrome Spinal canal stenosis Depression/depressive mood Other psychological conditions Anxiety disorders Burnout Cancer Breast cancer Thyroid cancer Other conditions: skin diseases (psoriasis, neurodermatitis), scoliosis, hip dysplasia, abdominal hernia, neurological disorders (multiple sclerosis, fatigue syndrome), allergies/hay fever, Factor V disorder, post-Coronavirus Disease syndrome, migraines, tinnitus, lymphatic diseases (lymphedema), fibromyalgia		51.8 % (44) 21.8 % (19) 27.1 % (23) 30.5 % (25) 10.7 % (9) 7.0 % (6) 5.8 % (5) 9.3 % (8) 9.4 % (8) 16.7 % (14) 4.6 % (4) 17.2 % (15) 13.8 % (12) 5.9 % (5) 20.5 % (17) 10.7 % (9) 25.0 % (21) 9.5 % (8) 18.6 % (16) 3.6 % (3) 1.2 % (1) 9.6 % (8) 22.1 % (19) 5.3 % (4) 5,3 % (4) 11.8 % (10) 6.0 % (5) 20.0 % (17) 11.1 % (9) 6.3 % (5) 2.5 % (2) 7.0 % (6) 5.8 % (5) 1.2 % (1) 17.2 % (15)
**Physical examination**		baseline population
**Auscultation of Thorax** Rhythmic hear rhythm Clear heart sound Additional heart sounds / others Diminished breath sounds Stridor / obstructive breath sounds		100 % (96) 96.9 % (93) Systolic (3rd intercostal room left) (1), tachycardia (1), paroxysmal atrial fibrillation (1) 1.0 % (1) 2.4 % (2) / 2.4 % (2)
**Abdominal Examination** Soft / distended abdominal wall tenderness Normal / pathological bowel sounds Regular bowel history Constipation / Diarrhea / irregular		98.9 % (94) / 1.1 % (1) 2.1 % (2) 98.9 % (94) / 1.1 % (1) 77.8 % (70) 8.9 % (8) / 10.0 % (9) / 3.3 % (3)
**Neurological Examination** Symmetric / increased / decrease muscle tone Unremarkable strength assessment Intact / pathological sensation		96.8 % (90) / 2.2 % (2) / 1.1 % (1) 100 % (85) 81.5 % (75) / 18.5 % (17)
**Family medical history** Overweight/obesity in the family Diabetes mellitus in the family Hypertension in the family Dyslipidemia in the family Myocardial infarction/sudden cardiac death in the family Arteriosclerosis/vascular occlusion in the family Stroke in the family Kidney disease in the family Depression in the family Cancer in the family		81.2 % (69) 52.3 % (46) 76.5 % (65) 22.5 % (18) 44.2 % (38) 44.4 % (36) 29.8 % (25) 26.2 % (22) 29.3 % (24) 52.3 % (46)
**Joint complaints**		baseline population
General physical complaints		82.4 % (85)
Joint complaints in the past 12 months		83.9 % (87)
Joint complaints in the last 24 h		51.2 % (86)
Pain in the last 24 h Mild/Moderate/Severe	Shoulder	Left side: 11.2 % / 7.1 % / 0.0 % (98) Right side: 13.3 % / 5.1 % / 1.0 % (98)
	Elbow	Left side: 4.1 % / 1.0 % / 0.0 % (98) Right side: 6.1 % / 1.0 % / 1.0 % (98)
	Wrist	Left side: 6,1 % / 3,1 % / 0,0 % (98) Right side: 4.1 % / 5.1 % / 0.0 % (98)
	Finger joints	Left side: 13.3 % / 4.1 % / 0.0 % (98) Right side: 11.2 % / 4.1 % / 1.0 % (98)
	Hip	Left side: 8.2 % / 7.1 % / 3.1 % (98) Right side: 10.2 % / 4.1 % / 5.1 % (98)
	Knee	Left side: 11.2 % / 14.3 % / 1.0 % (98) Right side: 13.3 % / 14.3 % / 4.1 % (98)
	Ankle	Left side: 10.2 % / 9.2 % / 0.0 % (98) Right side: 6.1 % / 11.2 % / 0.0 % (98)
	Toe joints	Left side: 6.1 % / 4.1 % / 0.0 % (98) Right side: 6.1 % / 3.1 % / 0.0 % (98)
Signs of inflammation (top) Full mobility (bottom)	Shoulder	Left side: 1.1 % (93) / Right side: 0.0 % (93)
		Left side: 92.0 % (87) / Right side: 93.0 % (86)
	Elbow	Left side: 0.0 % (93) / Right side: 0.0 % (92)
		Left side: 98.8 % (83) / Right side: 98.8 % (83)
	Wrist	Left side: 0.0 % (94) / Right side: 0.0 % (93)
		Left side: 96.4 % (84) / Right side: 95.2 % (84)
	Hip	Left side: 1.1 % (92) / Right side: 0.0 % (92)
		Left side: 83.3 % (84) / Right side: 86.7 % (83)
	Knee joint	Left side: 3.3 % (92) / Right side: 4.3 % (93)
		Left side: 88.0 % (83) / Right side: 89.3 % (84)
	Ankle joint	Left side: 0.0 % (93) / Right side: 0.0 % (93)
		Left side: 100 % (83) / Right side: 100 % (83)
Pain (top) Movement restriction (bottom)	Cervical spine (C-spine)	13.3 % (90)
		15.9 % (88)
	Thoracic spine (T-spine)	4.4 % (90)
		11.4 % (88)
	Lumbar spine (L-spine)	29.8 % (94)
		33.7 % (89)

^1^BMI, body mass index

Physical examination showed normal heart rhythm in all participants, although 2.4 % exhibited abnormal respiratory sounds. Abdominal distension (1.1 %), tenderness (2.1 %), constipation (8.9 %) and diarrhea (10.0 %) were reported. Neurological assessments demonstrated symmetrical muscle tone in 96.8 % and intact sensory function in 81.5 %. In terms of family history, 81.2 % of participants reported a familial predisposition to overweight or obesity. Hypertension was also prevalent within families, affecting 76.5 % of participants' relatives. Moreover, 52.3 % had a family history of diabetes mellitus, and 44.2 % of heart disease, including myocardial infarction or sudden cardiac death. Notably, 29.3 % had a family history of depression, and 52.3 % reported cancer among family members, further highlighting the genetic and environmental risk factors for both metabolic and psychological conditions within this population (see Table 4).

### 3.3 Baseline musculoskeletal health

Chronic joint pain was common, with 82.4 % of participants reporting musculoskeletal discomfort, and 83.9 % experiencing joint pain over the past year (see Table 4). Additionally, 51.2 % had joint pain in the 24 h prior to assessment. Pain was primarily concentrated in the knees, hips, and shoulders. Mild knee pain was reported by 11.2 % (left) and 13.3 % (right) of participants, while moderate pain affected 14.3 % for both knees. Shoulder pain was present in 18.3 %, and hip pain affected 15.3 % (left) and 14.3 % (right). Elbow, wrist, and finger pain were less common, and ankle and toe joints were minimally impacted. Inflammation was most frequent in the knees (3.3 % left, 4.3 % right), with minimal signs in the shoulders and hips. Mobility remained largely intact for most joints, with over 90 % retaining full movement in shoulders, wrists, elbows, and ankles. However, mobility restrictions were more evident in the hips (16.7 % left, 13.3 % right) and knees (12.0 % left, 10.7 % right), while the lumbar spine showed the greatest impairment, with 29.8 % reporting pain and 33.7 % noting restricted movement.

## 4 Discussion

Obesity poses a global health challenge necessitating a multifaceted approach. Our trial examines evidence supporting long-term obesity management, emphasizing a personalized, holistic, general practitioner-centered approach in rural areas. By addressing the unique socio-cultural and geographical challenges faced by these populations, HAPpEN leverages general practitioners as key facilitators recognizing various contributing factors such as genetics, metabolism, medical history, lifestyle, and psychosocial elements (Ahima and Lazar, 2013). Continuous education and support provided by general practitioners have been shown to empower patients, enhancing health literacy and fostering sustained improvements in weight management (Burke et al., 2011; Moore et al., 2019). HAPpEN's general practitioner-centered therapy integrates dietary and physical activity guidance, and evidence-based behavioral interventions, ensuring that general practitioners are equipped to deliver personalized, non-judgmental care effectively, seen to be lacking in the past (Rubino et al., 2021). Moreover, this trial aligns with existing literature on weight history, motivators, and barriers to weight loss, while providing additional insight into rural settings. A high prevalence of childhood overweight reinforces previous findings linking early obesity to adulthood, while decreased exercise and poor dietary habits emerge as significant contributors to weight gain (Simmonds et al., 2016). Motivators such as improved well-being and physical health echo results from other studies. Additionally, the high rate of weight regain reflects the complexities of long-term weight management and the associated challenges, including lack of physical activity, difficulties with eating behaviors and psychological factors (Trujillo-Garrido and Santi-Cano, 2022). Our data capture district-level rural health characteristics absent from broader datasets (Mensink et al., 2013). This nuanced profile supports tailored health interventions in rural settings.

Importantly, the program incorporates smartphone-based tools, which have proven to enhance communication and flexibility among patients and therapists, by fostering interdisciplinary collaboration peer support and improve empowerment, knowledge, adherence and clinical outcomes at reduced costs (Burke et al., 2011; Ufholz and Werner, 2023). Our trial introduces a smartphone-based, stepwise self-monitoring diary for tracking dietary habits and physical activity while emphasizing motivational and self-regulatory factors essential for sustainable lifestyle changes (Palmeira et al., 2023). This supports the comprehensive and personalized approach to obesity management, especially for rural areas, with limited health-related resources. In this context, the formative survey conducted in rural Kulmbach was instrumental in addressing the region-specific intervention needs.

The baseline data of the HAPpEN cohort indicates a high prevalence of obesity and comorbidities, with a higher average BMI and greater proportion of participants with grade III obesity, highlighting the program's success in engaging patients with significant treatment needs.

Our data provide insights into challenges of obesity management in German rural settings. Given the structural weakness in rural healthcare, including a shortage of nutritionists and psychotherapists, HAPpEN maximizes available resources by having general practitioners take on a central advisory role, that they have inherent by nature from patient's perspective (Wangler and Jansky, 2023). Additionally, HAPpEN acknowledges the fact that fighting obesity is more effective in like-minded groups (Street and Avenell, 2022) by offering sporting groups, a digital forum to exchange efforts or recipes and foster participant exchanges to enhance self-efficacy and learning.

The flexibility of the pragmatic approach in HAPpEN allows for intervention adaptation and strategy identification, though the lack of a non-intervention group may limit precise impact assessment and the ability to attribute observed changes in outcomes directly to HAPpEN. The program also primarily targets patients with a BMI ≥ 30 kg/m (Blüher, 2019), excluding overweight individuals who may benefit from early intervention.

## 5 Conclusion

In conclusion, HAPpEN aims to enhance health literacy and motivation among rural residents through interdisciplinary, evidence-based obesity management, focusing on behavioral interventions seen to be crucial in the past (McEwan et al., 2016). A formative survey and baseline sample characteristics underscore the need for treatment options in rural areas. General practitioners, skilled in complex conversations and long-term patient contact, are well-suited for this role. Anticipated benefits include improved health, enhanced patient knowledge and reduced healthcare utilization. Evaluating implementation, clinical outcomes, and economic impacts will inform policy and guideline. General practitioner-led interventions like HAPpEN may inspire broader adoption, while future research should explore sustainability, digital tools, and social networks. Balancing effort and health system benefits remain a long-term challenge.

## Declaration of generative Artificial Intelligence and Artificial Intelligence-assisted technologies in the writing process

Preparing this manuscript, the authors used ChatGPT to improve the English writing and phrasing. After using this tool, the authors reviewed and edited the content as needed and take full responsibility for the content of the published article.

## Funding statement

This work was supported by the Bavarian Federal Ministry of Health, Care and Prevention (G31i-G8000–2021/2630–23, 07.06.2022).

## CRediT authorship contribution statement

**Marika Haderer:** Writing – original draft, Visualization, Project administration, Methodology, Funding acquisition, Data curation, Conceptualization. **Reiner Hofmann:** Writing – review & editing, Supervision, Resources, Funding acquisition, Conceptualization. **Tina Bartelmeß:** Writing – review & editing, Supervision, Methodology, Conceptualization. **Laura König:** Writing – review & editing, Supervision, Methodology, Conceptualization. **Constanze Betz:** Writing – review & editing, Methodology, Data curation. **Mirna Al Masri:** Writing – review & editing, Methodology. **Alisa Bader:** Writing – review & editing, Methodology. **Natascha von Schau:** Writing – review & editing, Supervision, Methodology, Funding acquisition, Conceptualization.

## Declaration of competing interest

The authors declare that they have no known competing financial interests or personal relationships that could have appeared to influence the work reported in this paper.

## Data Availability

Data will be made available on request.

## Appendix A Supplementary data

Supplementary material Image 1

## References

[bb0005] Ahima R.S., Lazar M.A. (2013). Physiology. The health risk of obesity - Better metrics imperative. Science.

[bb0010] Barrett S., Begg S., O’Halloran P., Kingsley M. (2018). Integrated motivational interviewing and cognitive behaviour therapy for lifestyle mediators of overweight and obesity in community-dwelling adults: a systematic review and meta-analyses. BMC Public Health.

[bb0015] BBSR (2024). Raumbeobachtung - Siedlungsstruktureller Typ von Stadt-Land-Regionen. Accessed December 22. https://www.bbsr.bund.de/BBSR/DE/forschung/raumbeobachtung/Raumabgrenzungen/deutschland/regionen/siedlungsstrukturtypen-stadt-land-regionen/StadtLandRegionen_Typen.html.

[bb0020] Berg A., Frey I., Hamm M. (2008). Das M.O.B.I.L.I.S.-Programm. Adipositas - Ursachen, Folgeerkrankungen. Ther.

[bb0025] Berghöfer A., Pischon T., Reinhold T., Apovian C.M., Sharma A.M., Willich S.N. (2008). Obesity prevalence from a European perspective: a systematic review. BMC Public Health.

[bb0030] Beverly E.A. (2024). Obesity management solutions in rural communities. Curr. Cardiovasc. Risk Rep..

[bb0035] Blüher M. (2019). Obesity: global epidemiology and pathogenesis. Nat. Rev. Endocrinol..

[bb0040] Boehm B.O., Claudi-Boehm S., Yildirim S. (2005). Prevalence of the metabolic syndrome in Southwest Germany. Scand. J. Clin. Lab. Investig. Suppl..

[bb0045] Borders T.F., Rohrer J.E., Cardarelli K.M. (2006). Gender-specific disparities in obesity. J. Community Health.

[bb0050] Borek A.J., Abraham C., Greaves C.J. (2019). Identifying change processes in group-based health behaviour-change interventions: development of the mechanisms of action in group-based interventions (MAGI) framework. Health Psychol. Rev..

[bb0055] Bull F.C., Maslin T.S., Armstrong T. (2009). Global physical activity questionnaire (GPAQ): nine country reliability and validity study. J. Phys. Act. Health.

[bb0060] Burke L.E., Wang J., Sevick M.A. (2011). Self-monitoring in weight loss: a systematic review of the literature. J. Am. Diet. Assoc..

[bb0065] Carrillo-Álvarez E., Kawachi I., Riera-Romaní J. (2019). Neighbourhood social capital and obesity: a systematic review of the literature. Obes. Rev..

[bb0070] Cheah W.H., Mat Jusoh N., Aung M.M.T., Ab Ghani A., Rebuan Mohd Amin, H. (2022). Mobile Technology in Medicine: development and validation of an adapted system usability scale (SUS) questionnaire and modified technology acceptance model (TAM) to evaluate user experience and acceptability of a Mobile application in MRI safety screening. Indian J. Radiol. Imaging..

[bb0075] Durrer Schutz D., Busetto L., Dicker D. (2019). European practical and patient-Centred guidelines for adult obesity management in primary care. Obes. Facts.

[bb0080] (2017). Bundeszentrale für gesundheitsliche Aufklärung (BZgA). Nationale Empfehlungen für Bewegung und Bewegungsförderung. *Forschungs und Prax der Gesundheitsförderung*. Published online.

[bb0085] Göhner W., Schlatterer M., Seelig H., Frey I., Berg A., Fuchs R. (2012). Two-year follow-up of an interdisciplinary cognitive-behavioral intervention program for obese adults. Aust. J. Psychol..

[bb0090] Hauner H., Moss A., Berg A. (2014). Interdisziplinäre Leitlinie der Qualität S3 zur “Prävention und Therapie der Adipositas”. Adipositas - Ursachen, Folgeerkrankungen, Ther..

[bb0095] König D., Hörmann J., Predel H.G., Berg A.A. (2018). A 12-month lifestyle intervention program improves body composition and reduces the prevalence of prediabetes in obese patients. Obes. Facts.

[bb0100] König L.M., Betz C., Al Masri M., Bartelmeß T. (2024). Data note: health behaviors and mobile intervention use in patients recruited from general practitioners’ practices in rural Bavaria. Published online.

[bb0105] Küpper P. (2016).

[bb0110] Laugwitz B., Held T., Schrepp M. (2008). Lecture Notes in Computer Science.

[bb0115] Lim S., Lee W.K., Tan A. (2021). Peer-supported lifestyle interventions on body weight, energy intake, and physical activity in adults: a systematic review and meta-analysis. Obes. Rev..

[bb0120] McEwan D., Harden S.M., Zumbo B.D. (2016). The effectiveness of multi-component goal setting interventions for changing physical activity behaviour: a systematic review and meta-analysis. Health Psychol. Rev..

[bb0125] Mensink G.B.M., Schienkiewitz A., Haftenberger M., Lampert T., Ziese T., Scheidt-Nave C. (2013). Übergewicht und Adipositas in Deutschland: Ergebnisse der Studie zur Gesundheit Erwachsener in Deutschland (DEGS1). Bundesgesundheitsblatt.

[bb0130] Michie S., Wood C.E., Johnston M., Abraham C., Francis J.J., Hardeman W. (2015). Behaviour change techniques: the development and evaluation of a taxonomic method for reporting and describing behaviour change interventions a suite of five studies involving consensus methods, randomised controlled trials and analysis of qualitative da. Health Technol Assess (Rockv)..

[bb0135] Moore S.E., McMullan M., McEvoy C.T., McKinley M.C., Woodside J.V. (2019). The effectiveness of peer-supported interventions for encouraging dietary behaviour change in adults: a systematic review. Public Health Nutr..

[bb0140] Morfeld M., Bullinger M. (2008). Der SF-36 Health Survey zur Erhebung und Dokumentation gesundheitsbezogener lebensqualität. Phys Medizin, Rehabil Kurortmedizin..

[bb0145] Nackers L.M., Dubyak P.J., Lu X., Anton S.D., Dutton G.R., Perri M.G. (2015). Group dynamics are associated with weight loss in the behavioral treatment of obesity. Obesity (Silver Spring).

[bb0150] (NCD-RisC)., NCD Risk Factor Collaboration (2021). Heterogeneous contributions of change in population distribution of body mass index to change in obesity and underweight. Elife.

[bb0155] Palmeira A.L., Marques M.M., Sánchez-Oliva D. (2023). Are motivational and self-regulation factors associated with 12 months’ weight regain prevention in the NoHoW study? An analysis of European adults. Int. J. Behav. Nutr. Phys. Act..

[bb0160] Pfeiffer C., Robitzsch A., Teufel M., Skoda E.-M. (2020). Diagnostik von Essstörungen bei Adipositas. CardioVasc.

[bb0165] Quintiliani L.M., DeBiasse M.A., Branco J.M., Bhosrekar S.G., Rorie J.A.L., Bowen D.J. (2014). Enhancing physical and social environments to reduce obesity among public housing residents: rationale, trial design, and baseline data for the healthy families study. Contemp. Clin. Trials.

[bb0170] Rubino F., Logue J., Bøgelund M. (2021). Attitudes about the treatment of obesity among healthcare providers involved in the care of obesity-related diseases: a survey across medical specialties in multiple European countries. Obes. Sci. Pract..

[bb0175] Rudolph A., Hellbardt M., Baldofski S., De Zwaan M., Hilbert A. (2016). Evaluation des einjährigen multimodalen Therapieprogramms DOC WEIGHT® 1.0 zur Gewichtsreduktion bei Patienten mit Adipositas Grad II und III. Psychother. Psychosom. Med. Psychol..

[bb0180] Schlesinger S., Neuenschwander M., Schwedhelm C. (2019). Food groups and risk of overweight, obesity, and weight gain: a systematic review and dose-response Meta-analysis of prospective studies. Adv. Nutr..

[bb0185] Sekhon M., Cartwright M., Francis J.J. (2022). Development of a theory-informed questionnaire to assess the acceptability of healthcare interventions. BMC Health Serv. Res..

[bb0190] Simmonds M., Llewellyn A., Owen C.G., Woolacott N. (2016). Predicting adult obesity from childhood obesity: a systematic review and meta-analysis. Obes. Rev..

[bb0195] Spitzer R.L., Kroenke K., Williams J.B.W. (1999). Validation and utility of a self-report version of PRIME-MD: the PHQ primary care study. Primary care evaluation of mental disorders. Patient health questionnaire. JAMA.

[bb0200] Statistische Bibliothek (2022).

[bb0205] Stelmach-Mardas M., Rodacki T., Dobrowolska-Iwanek J. (2016). Link between food energy density and body weight changes in obese adults. Nutrients.

[bb0210] Street S., Avenell A. (2022). Are individual or group interventions more effective for long-term weight loss in adults with obesity? A systematic review. Clin Obes..

[bb0215] Trivedi T., Liu J., Probst J., Merchant A., Jones S., Martin A.B. (2015). Obesity and obesity-related behaviors among rural and urban adults in the USA. Rural Remote Health.

[bb0220] Trujillo-Garrido N., Santi-Cano M.J. (2022). Motivation and limiting factors for adherence to weight loss interventions among patients with obesity in primary care. Published online.

[bb0225] Ufholz K., Werner J. (2023). The efficacy of Mobile applications for weight loss. Curr. Cardiovasc. Risk Rep..

[bb0230] Van Dijk L., Otters H.B., Schuit A.J. (2006). Moderately overweight and obese patients in general practice: a population based survey. BMC Fam. Pract..

[bb0235] Wangler J., Jansky M. (2023). How are people with obesity managed in primary care? – results of a qualitative, exploratory study in Germany 2022. Arch. Public Health.

[bb0240] Westenhöfer J., Fintelmann S., Sievers C. (2003). Progress in Obesity Research.

[bb0245] Whitlock E.P., Orleans C.T., Pender N., Allan J. (2002). Evaluating primary care behavioral counseling interventions. An evidence-based approach. Am. J. Prev. Med..

[bb0250] World Health Organization (2020).

