# Supplementary material for: General practitioner-centered rural obesity management: Design, protocol and baseline data of the German HAPpEN pragmatic trial
Source: Prev Med Rep. 2024 Dec 26;49:102959. doi: 10.1016/j.pmedr.2024.102959 (PMC11755079; doi:10.1016/j.pmedr.2024.102959)
Supplement: Supplementary file 1 — Supplementary material [file mmc1.docx]

**General Practitioner-centered rural obesity management: design, protocol and baseline data of the German HAPpEN pragmatic trial**

***Formative Survey:***

The formative survey to the HAPpEN trial design covered socio-demographic information, health status, lifestyle, information sources, and technology use^1^.The informative pre-test found a population predominantly female (52%) with an average age of 51.3 ± 16.6. While the mean body mass index (BMI) amongst participants was 28.3 ± 6.6, a total of 35.7% was classified overweight and 29.8% as obese (a total of 65.5% either overweight or obese) (see Supplementary Table 1 - 3). The highest proportion of obese patients was among those aged 50 – 59 (86.2%), followed by 40 – 49 (81.6%) and 60 – 69 (73.2%), whereas overweight individuals ranked the following: 50 – 59 (25.6%), followed by 60 – 69 (16.7%) and 40 – 49 (14.4%). Gender discrepancies were noted, with females more often in the normal weight category (36.2%) or overweight (30.5%), while males were more frequently overweight (43.1%) or obese grade I (21.6%). Most meals, including breakfast (69.5%) and dinner (96.9%) were eaten at home, with 59.7% regularly cooking at home. Access to healthy food varied, with 56.4% finding it (somewhat) difficult. Exercise habits were generally low, with 73.8% never using a club and 62.4% never using a gym. Only 7% regularly exercised outside the home or in organized facilities. For almost 50% of participants, information sources were primarily the internet, followed by magazines, cookbooks, family, and social media. Social media was primarily used for seeking inspiration (62%). Smartphone ownership was high (91.4%), but only a third used fitness trackers or apps with Yazio, My Fitness Pal, Lifesum, and Weight Watchers being the most common.

Supplementary Table 1: Body Mass Index Category of 273 adults participating in the formative survey (December 2022 – February 2023) by Age.

| BMI^[[1]](#footnote-1)^ | | <18.5 | | 18.5-24.9 | | 25-29.9 | | 30-34.9 | | 35-39.9 | | >40 | | total | |
| --- | --- | --- | --- | --- | --- | --- | --- | --- | --- | --- | --- | --- | --- | --- | --- |
|  |  | underweight | | normal weight | | overweight | | obesity grade I | | obesity grade II | | obesity grade III | |  | |
|  |  | N | % | N | % | N | % | N | % | N | % | N | % | N | % |
| Age groups | 18-29 | 3 | 23.1% | 16 | 21.6% | 6 | 6.7% | 7 | 14.9% | 1 | 6.3% | 0 | 0.0% | 33 | 13.1% |
|  | 30-39 | 1 | 7.7% | 9 | 12.2% | 15 | 16.7% | 5 | 10.6% | 0 | 0.0% | 0 | 0.0% | 30 | 11.9% |
|  | 40-49 | 1 | 7.7% | 6 | 8.1% | 13 | 14.4% | 8 | 17.0% | 5 | 31.3% | 4 | 33.3% | 37 | 14.7% |
|  | 50-59 | 1 | 7.7% | 24 | 32.4% | 23 | 25.6% | 17 | 36.2% | 4 | 25.0% | 3 | 25.0% | 72 | 28.6% |
|  | 60-69 | 4 | 30.8% | 9 | 12.2% | 15 | 16.7% | 7 | 14.9% | 4 | 25.0% | 4 | 33.3% | 43 | 17.1% |
|  | 70-79 | 0 | 0.0% | 10 | 13.5% | 13 | 14.4% | 2 | 4.3% | 2 | 12.5% | 1 | 8.3% | 28 | 11.1% |
|  | >80 | 3 | 23.1% | 0 | 0.0% | 5 | 5.6% | 1 | 2.1% | 0 | 0.0% | 0 | 0.0% | 9 | 3.6% |
| total | | 13 | 100.0% | 74 | 100.0% | 90 | 100.0% | 47 | 100.0% | 16 | 100.0% | 12 | 100.0% | 252 | 100.0% |

Supplementary Table 2: Body Mass Index Category of 273 adults participating in the formative survey (December 2022 – February 2023) by Gender.

| BMI^[[2]](#footnote-2)^1 category | male | | female | | diverse | | total | |
| --- | --- | --- | --- | --- | --- | --- | --- | --- |
|  | N | % | N | % | N | % | N | % |
| <18.5 | 4 | 3.4% | 9 | 6.4% | 0 | 0.0% | 13 | 4.8% |
| 18.5-24.9 | 25 | 21.6% | 51 | 36.2% | 0 | 0.0% | 80 | 29.4% |
| 25.0-29.9 | 50 | 43.1% | 43 | 30.5% | 0 | 0.0% | 95 | 34.9% |
| 30.0-34.9 | 25 | 21.6% | 22 | 15.6% | 1 | 100.0% | 53 | 19.5% |
| 35.0-39.9 | 6 | 5.2% | 10 | 7.1% | 0 | 0.0% | 18 | 6.6% |
| >40.0 | 6 | 5.2% | 6 | 4.3% | 0 | 0.0% | 13 | 4.8% |
| total | 116 | 100.0% | 141 | 100.0% | 1 | 100.0% | 272 | 100.0% |

Supplementary Table 3: Key Findings from the formative survey of 273 adults in Kulmbach District (December 2022 – February 2023) informing the HAPpEN trial.

| Formative Survey Findings | Conclusion | Realization in HAPpEN |
| --- | --- | --- |
| High prevalence of overweight (35.7%) and obesity (29.8%) in rural Germany (mean BMI^[[3]](#footnote-3)^1: 28.3 ± 6.6) | High obesity prevalence in rural Germany necessitating targeted interventions | General practitioner-centered approach for rural areas focusing on patients with obesity, targeting them lowthreshold through their already existing care office |
| Highest proportion of obesity (86.2%) in patients aged 50 – 59 years, followed by 40 – 49 years (81.6 %) and 60 – 69 years (73.2%) | Higher prevalence of obesity in patients ≥ 40 years | trail included patients up to 65 years, requiring age-appropriate, accessible interventions: 1. Paper-pencil survey design 2. HAPpEN app with streamlined functionality for improved user experience |
| More male patients with overweight and obesity (64.7%) | Higher rates of overweight and obesity among men necessitating targeted motivation strategies for effective management and prevention | interventions included hiking events and muscle-strengthening activities guided by physiotherapists.  A dedicated men-only exercise group was established, starting at higher intensity levels with shorter progression intervals. Optional integration of weight training. |
| High response rate (80.5%) | Survey utilized a paper-pencil design to ensure high response rates and accessibility | Survey distributed by general practitioners during check-ups and waiting periods to maximize convenience - design accounted for older participants |
| Preference for home-cooked meals consumed at home | Importance of culinary skills in intervention | 1. initial training on health-promoting food and by 2. General practitioner-led monthly nutritional counseling 3. Recipe sharing facilitated via the HAPpEN homepage and forum, 4. (App-linked) food-related challenges 5. Organized cooking and barbecue events |
| Reported challenges in accessing suitable food options (restaurants) in rural areas | Limited access to suitable food options in rural areas | Exchange of restaurant and dining recommendations in the vicinity encouraged through HAPpEN forum |
| Low exercise habits | Promotion of physical activity in context of obesity management | 1. initial training on health-promoting, and by 2.General practitioner-led monthly teaching session, 3. Physiotherapist-led exercise group sessions, held near participants’ homes. 4. Option for home practice through animated app-based tutorials (see Figure 2) |
| primary digital information sources, high ownership rate of smartphones but limited use of tracking apps | Digital support tools integrated as a key intervention component | HAPpEN homepage and forum for participant engagement, HAPpEN app designed without food tracking, instead featuring a step-wise grading scale for nutrition and physical activity (see Supplementary Table 5) |

Supplementary Table 4. General Practitioner-led Guideline-Compliant Obesity Management in the pragmatic HAPpEN trial.^[[4]](#footnote-4)^

| S3 Guideline Obesity Prevention and Therapy | Implementation in pragmatic HAPpEN trail |
| --- | --- |
| Initial Examination: medical history: weight and family history, previous therapy attempts, dietary habits and eating behavior, physical activity, motivation, psychosocial history  Examinations: body height and weight, waist circumference, blood pressure, laboratory testing: fasting blood sugar, Hemoglobin A1c, oral glucose tolerance test, total cholesterol, high-density-, and low-density-lipoprotein-cholesterol, triglycerides, uric acid, creatinine, electrolytes, thyroid-stimulating hormone, microalbuminuria or albumin/creatinine ratio in urine, electrocardiogram, ergometry∗, echocardiography∗, 24-hour blood pressure monitoring∗, sleep apnea screening∗, upper abdominal sonography∗, doppler sonography∗ (*optional examination) | Initial Examination: medical history via questionnaire: social anamnesis, nutritional and exercise behaviors and literacy, weight history, joint complaints, sleep habits, family history, wellbeing, quality of life  Examinations: body height, weight, waist circumference, blood pressure, heart rate, joint function, thoracic auscultation, abdominal examination, motor and sensory tests, ergometry, electrocardiogram, laboratory analysis: Hemoglobin A1c, C-reactive protein, uric acid, liver function tests, total cholesterol, high-density- and low-density-lipoprotein-cholesterol, thyroid-stimulating hormone, creatinine and estimated glomerular filtration rate, complete blood count |
| follow-up examinations may also be necessary. The content and frequency of a follow-up examination depend on the results of the initial examination and the course of therapy. The follow-up examinations serve to prevent complications that may arise due to weight loss. The following parameters should be assessed, if necessary: medical history, body weight, nutrition, eating behavior, physical activity, motivation for lifestyle change.  Examinations: heart rate, blood pressure, anthropometry: body weight, waist circumference; laboratory tests (depending on initial findings): fasting blood sugar, Hemoglobin A1c, total cholesterol, high-density- and low-density-lipoprotein-cholesterol, triglycerides, Glutamate Pyruvate Transaminase, uric acid, creatinine, electrolytes | follow-up examinations using surveys and examinations at six and 12 months: questionnaire on nutrition and eating behavior and literacy, exercise history, joint complaints, medication use, sleep habits, wellbeing, quality of life  Examinations: body height, weight, waist circumference, blood pressure, heart rate, joint function, thoracic auscultation, abdominal examination, motor and sensory tests, ergometry, electrocardiogram, laboratory analysis: Hemoglobin A1c, C-reactive protein, uric acid, liver function tests, total cholesterol, high-density- and low-density-lipoprotein-cholesterol, creatinine and estimated glomerular filtration rate, complete blood count |
| Motivation, readiness, and resources of patients for lifestyle change should be assessed for each individual component of therapy: nutrition, exercise, and behavioral therapy | Using monthly appointments with the patients, motivation, readiness, and resources are regularly assessed by general practitioners |
| Obesity management basis program including nutrition, exercise and behavior change - nutritional therapy and physical activity should be personalized, tailored to therapy and risk profiles  Behavioral therapy involves interventions in individual or group settings, ensuring individual adaptation based on past experiences and social conditions such as partners, family, friends, workplace, and leisure activities.  It integrates the following psychotherapeutic elements:   - Self-monitoring of behavior and progress (body weight, food intake, exercise) → Keeping journals helps highlight specifics - Practicing flexible control of eating and exercise behavior - Stimulus control: Learning strategies for dealing with food - Cognitive restructuring: Dysfunctional thoughts are identified through interviews and by keeping self-monitoring journals - Social competence/assertiveness training - Social support | Recognized nutritional counseling by general practitioners as well as sport group therapy by certified physiotherapists. Doctors are trained for behavior change techniques and will use them in the monthly appointments.  Behavioral therapy, personalized nutritional and exercise counseling through monthly appointments. Social support through exchange with other affected individuals during physiotherapy session, training and cooking as well as hiking events. A digital forum was provided for patients and social support and networking was analyzed using interviews and surveys. |
| On-site programs can also be synergistically supplemented by online tools (e.g., apps for monitoring food intake and physical activity). | App as digital self-monitoring diary for monitoring weight loss progress, and for home-based exercise using animated tutorials |

Supplementary Table 5: Multi-Stage Grading Scale for Dietary Recommendations and Physical Activity, Evidence-Based and Guideline-Compliant^2–8^.^[[5]](#footnote-5)^3

|  | | | |
| --- | --- | --- | --- |
| 1. Energy-providing beverages | | 1. evening meals | |
| Minimum | Becoming aware of the amount of sugary soft drinks consumed | Minimum | Becoming aware of evening meals (especially after seven/eight pm) |
| Level 1 | max. one glass (0.25 l) /day | Level 1 | No eating after seven/eight pm or at least three hours before bedtime one to two days/ week |
| Level 2 | max. four to five glasses /week | Level 2 | No eating after seven/eight pm or at least three hours before bedtime three to four days/ week |
| Level 3 | max. three glasses /week or only on weekends | Level 3 | No eating after seven/eight pm five days/week |
| Maximum | complete abstention or up to max. two glasses /week | Maximum | No eating after seven/eight pm six to seven days/week |
| 1. Snacks | | 9a. Whole grain product | |
| Minimum | Becoming aware of the number of snacks consumed, alternatively drinking a glass of water | Minimum | Becoming aware: What whole grain products are available? Where could I implement whole grain products in my diet? |
| Level 1 | Reduction of frequency: max. one/day | Level 1 | Replace white flour with whole grain products in one to two meals/week |
| Level 2 | max. four times/week | Level 2 | Replace white flour with whole grain products in three to four meals/week |
| Level 3 | max. two to three times/week | Level 3 | Replace white flour with whole grain products five to six meals/week |
| Maximum | once or no sweets/week | Maximum | Replace all white flour with whole grain products |
| 1. Fast Food | | 9b. Fruit and Vegetables | |
| Minimum | Becoming aware of the consumption of fast food per week | Minimum | Becoming aware: How much sugar/fiber does each fruit/vegetable have? |
| Level 1 | max. four times/week | Level 1 | one piece of vegetable or fruit/day |
| Level 2 | max. two to three times/week | Level 2 | Two to three pieces of vegetables or fruit/day |
| Level 3 | max. two times/week | Level 3 | four pieces of vegetables or fruit/day |
| Maximum | no fast food or max. once/week | Maximum | five pieces of vegetables or fruit/day, preferably more vegetables (three times) than fruits (two times) |
| 1. In-between meals | | 9c. Proteins | |
| Minimum | Developing awareness of in-between meals consumption, alternatively drinking one glass of water | Minimum | Getting to know various protein sources  (animal, plant-based) as alternatives to meat |
| Level 1 | max. one to two in-between meals/day | Level 1 | Five to six meals with meat/week |
| Level 2 | finding healthy alternatives on four days/week | Level 2 | Three to four meals with meat/week |
| Level 3 | finding healthy alternatives on five to seven days/week | Level 3 | Two meals with meat/week |
| Maximum | taking three to four hours between meals, or having one healthy in-between meals/day | Maximum | maximum one meal with meat/week |
| 1. Frequence of meals | | 10. High/Low Fat consumption | |
| Minimum | Recognizing one's own rhythm | Minimum | Pay attention to hidden fats (e.g., in salad dressings) |
| Level 1 | One to two days/week with three main meals | Level 1 | Use rapeseed and olive oil as kitchen all-rounders |
| Level 2 | Three to four days/week with three main meals | Level 2 | Use low-fat options for one to two days/week |
| Level 3 | five days/week with three main meals | Level 3 | Use low-fat options for three to four days/week |
| Maximum | three main meals: breakfast, lunch, and dinner six to seven times/week | Level 4 | Use low-fat options for five days/week (optional) |
|  |  | Maximum | Use low-fat options for six to seven days/week |
| 1. breakfast | | 11. dining culture | |
| Minimum | Understanding what constitutes a healthy breakfast | Minimum | Becoming aware of hunger vs. appetite using the hunger scale |
| Level 1 | Having a healthy breakfast one to two days/week | Level 1 | Eat mindfully for one to two days/week |
| Level 2 | Having a healthy breakfast three to four days/week | Level 2 | Eat mindfully for three to four days/week |
| Level 3 | Having a healthy breakfast five days/week | Level 3 | Eat mindfully for five days/week |
| Maximum | Establishing a healthy breakfast six to seven days/week | Maximum | Eat mindfully for six to seven days/week |
| 1. Main meal | |  | |
| Minimum | Considering the energy density when selecting foods, drinking one glass of water before each meal to prevent consuming a large amount of food |  |  |
| Level 1 | One to two healthy main meals/week |  |  |
| Level 2 | Three to four healthy main meals/week |  |  |
| Level 3 | Five healthy main meals/week |  |  |
| Maximum | Six to seven healthy main meals/week |  |  |

| Physical activity grading scale | | | |
| --- | --- | --- | --- |
| 1. Active Transport: Walking or biking | | 1. Number of days engaging in muscle-strengthening activity | |
| Minimum | Be aware of opportunities for active transportation | Minimum | Identify time slots and exercises for muscle-strengthening physical activity |
| Level 1 | Use active transportation options one to two days/week | Level 1 | muscle-strengthening physical activity one day/week |
| Level 2 | Use active transportation options three to four days/week | Level 2 | muscle-strengthening physical activity two days/week |
| Level 3 | Use active transportation options five to six days/week | Level 3 | muscle-strengthening physical activity three days/week |
| Maximum | Use active transportation options seven days/week | Level 4 | muscle-strengthening physical activity four days/week |
|  |  | Maximum | muscle-strengthening physical activity on at least five days/week |
| 2, Increase the number of steps taken daily | | 1. Interrupting prolonged sitting periods | |
| Minimum | Be aware of opportunities to increase daily step count in everyday life | Minimum | Observe current sitting behaviour |
| Level 1 | Walk at least 4000 steps daily | Level 1 | Interrupt a prolonged sitting period once a day with five minutes of movement (e.g., walking around the house) |
| Level 2 | Walk at least 5000 steps daily | Level 2 | Interrupt a prolonged sitting period at least twice a day with five minutes of movement (e.g., walking around the house) |
| Level 3 | Walk at least 6000 steps daily | Level 3 | Sit for no longer than 60 minutes continuously on one to two days/week |
| Level 4 | Walk at least 7000 steps daily | Level 4 | Sit for no longer than 60 minutes continuously on three to four days/week |
| Level 5 | Walk at least 8000 steps daily | Level 5 | Sit for no longer than 60 minutes continuously on five to six days/week |
| Maximum | Walk at least 9000 steps daily | Maximum | Do not sit for longer than 60 minutes continuously/ day |
| 1. Number of minutes spent in moderate-intensity physical activity | |  |  |
| Minimum | Monitor current moderate and higher-intensity physical activity |  |  |
| Level 1 | Spend at least 10 minutes in moderate-intensity physical activity on at least one day/week |  |  |
| Level 2 | Spend at least 20 minutes in moderate-intensity physical activity on at least one day/week |  |  |
| Level 3 | Spend at least 30 minutes in moderate-intensity physical activity on at least one day/week |  |  |
| Level 4 | Spend at least 30 minutes in moderate-intensity physical activity on two days/week |  |  |
| Level 5 | Spend at least 30 minutes in moderate-intensity physical activity on three days/week |  |  |
| Level 6 | Spend at least 30 minutes in moderate-intensity physical activity on four days/week |  |  |
| Maximum | Spend at least 30 minutes in moderate-intensity physical activity on five days/week |  |  |

1. König LM, Betz C, Al Masri M, Bartelmeß T. Data Note: Health behaviors and mobile intervention use in patients recruited from general practitioners’ practices in rural Bavaria. Published online 2024:1-22.

2. Quintiliani LM, DeBiasse MA, Branco JM, Bhosrekar SG, Rorie JAL, Bowen DJ. Enhancing physical and social environments to reduce obesity among public housing residents: Rationale, trial design, and baseline data for the healthy families study. *Contemp Clin Trials*. 2014;39(2):201-210. doi:10.1016/j.cct.2014.08.005

3. Quintiliani LM, Whiteley JA, Murillo J, et al. Community health worker-delivered weight management intervention among public housing residents: A feasibility study. *Prev Med Reports*. 2021;22:101360. doi:10.1016/J.PMEDR.2021.101360

4. Schlesinger S, Neuenschwander M, Schwedhelm C, et al. Food Groups and Risk of Overweight, Obesity, and Weight Gain: A Systematic Review and Dose-Response Meta-Analysis of Prospective Studies. *Adv Nutr*. 2019;10(2):205-218. doi:10.1093/advances/nmy092

5. Schwingshackl L, Schwedhelm C, Hoffmann G, et al. Food groups and risk of all-cause mortality: a systematic review and meta-analysis of prospective studies. *Am J Clin Nutr*. 2017;105(6):1462-1473. doi:10.3945/AJCN.117.153148

6. Fastenau J, Kolotkin RL, Fujioka K, Alba M, Canovatchel W, Traina S. A call to action to inform patient‐centred approaches to obesity management: Development of a disease‐illness model. *Clin Obes*. 2019;9(3):e12309. doi:10.1111/cob.12309

7. Mytton OT, Panter J, Ogilvie D. Longitudinal associations of active commuting with body mass index. *Prev Med (Baltim)*. 2016;90:1-7. doi:10.1016/J.YPMED.2016.06.014

8. Paluch AE, Bajpai S, Bassett DR, et al. Daily steps and all-cause mortality: a meta-analysis of 15 international cohorts. *Lancet Public Heal*. 2022;7(3):e219-e228. doi:10.1016/S2468-2667(21)00302-9

9. Hauner H, Moss A, Berg A, et al. Interdisziplinäre Leitlinie der Qualität S3 zur „Prävention und Therapie der Adipositas”. *Adipositas - Ursachen, Folgeerkrankungen, Ther*. 2014;08(04):179-221. doi:10.1055/s-0037-1618857

1. BMI, body mass index [↑](#footnote-ref-1)
2. [↑](#footnote-ref-2)
3. 1 BMI, Body Mass Index [↑](#footnote-ref-3)
4. Analysis executed additionally to S3 Guideline^9^ (2013) are red. [↑](#footnote-ref-4)
5. 3 Sleep behaviour as one additional topic is not shown here. [↑](#footnote-ref-5)
